# Arrhythmogenic effects of ultra-long and bistable cardiac action potentials

**DOI:** 10.1371/journal.pcbi.1008683

**Published:** 2021-02-16

**Authors:** Stewart Heitmann, Anton Shpak, Jamie I. Vandenberg, Adam P. Hill

**Affiliations:** 1 Victor Chang Cardiac Research Institute, Darlinghurst, NSW, Australia; 2 Victor Chang Cardiac Research Institute Innovation Centre, Darlinghurst, NSW, Australia; 3 St Vincent’s Clinical School, UNSW Sydney, Kensington, NSW, Australia; University of Virginia, UNITED STATES

## Abstract

Contemporary accounts of the initiation of cardiac arrhythmias typically rely on after-depolarizations as the trigger for reentrant activity. The after-depolarizations are usually triggered by calcium entry or spontaneous release within the cells of the myocardium or the conduction system. Here we propose an alternative mechanism whereby arrhythmias are triggered autonomously by cardiac cells that fail to repolarize after a normal heartbeat. We investigated the proposal by representing the heart as an excitable medium of FitzHugh-Nagumo cells where a proportion of cells were capable of remaining depolarized indefinitely. As such, those cells exhibit bistable membrane dynamics. We found that heterogeneous media can tolerate a surprisingly large number of bistable cells and still support normal rhythmic activity. Yet there is a critical limit beyond which the medium is persistently arrhythmogenic. Numerical analysis revealed that the critical threshold for arrhythmogenesis depends on both the strength of the coupling between cells and the extent to which the abnormal cells resist repolarization. Moreover, arrhythmogenesis was found to emerge preferentially at tissue boundaries where cells naturally have fewer neighbors to influence their behavior. These findings may explain why atrial fibrillation typically originates from tissue boundaries such as the cuff of the pulmonary vein.

## Author summary

Cardiac fibrillation is a medical condition where normal heart function is compromised as electrical activity becomes disordered. How fibrillation arises spontaneously is not fully understood. It is generally thought to be triggered by premature depolarization of the cardiac action potential in one or more cells. Those premature beats, known as after-depolarizations, subsequently initiate a self-sustaining rotor in the otherwise normal heart tissue. In this study, we propose an alternative mechanism whereby arrhythmias are initiated by cardiac cells that fail to repolarize of their own accord but still operate normally when embedded in functional heart tissue. We find that such cells can act as focal ectopic sources under appropriate conditions of inter-cellular coupling. Moreover, those cells are more prone to initiating arrhythmia when they are located on natural tissue boundaries. This may explain why atrial fibrillation typically originates from the site where the pulmonary vein attaches to the wall of the heart.

## Introduction

Cardiac arrhythmias, which impair the heart’s ability to pump blood, are a major cause of morbidity [1] and mortality [2]. Once initiated, arrhythmias are sustained by re-entrant rotors of electrical activity in the myocardium [3–6]. Contemporary theories of the genesis of arrhythmias are grounded in the concept of a trigger and a substrate [7]. The trigger for an arrhythmia is often attributed to spontaneous ectopic activity, such as early after-depolarizations (EADs) or delayed after-depolarizations (DADs), in one or more cardiomyocytes. Whereas the substrate refers to a state of the myocardium which is particularly susceptible to initiating and supporting reentry. At least three factors can contribute to making the myocardium more vulnerable to initiating an arrhythmia. First, topological defects in the heart that form anatomical re-entry circuits, such as AV nodal accessory pathways [8]. Second, reduced intercellular connectivity, as for example occurs in stretched atria with diffuse interstitial fibrosis [1]. And third, heterogeneity in the refractoriness of cardiac cells, which is associated with arrhythmias in long QT syndrome [7] and is thought to predispose the tissue to uni-directional block [9, 10].

In the normal heart, there are regional variations in action potential duration, for example the epicardial to endocardial gradients in the ventricle [11] and systematic regional differences in the atria [12]. It is generally thought that the repolarization duration of individual cells, whilst changing gradually between regions, all fall on a continuum within a relatively narrow physiological range [13, 14]. However, as repolarization of the cardiac action potential represents a delicate balance between inward and outward currents, small perturbations to individual currents can have significant effects on repolarization.

In a recent study of atrial myocytes isolated from both rabbit and human, Kettlewell and colleagues [15] showed that widening the voltage window where there is sustained L-type calcium current (*I*_*CaL*_) by as little as 6 mV, can result in extreme changes in action potential duration and in some instances the cells fail to repolarize. Their findings are consistent with earlier simulation studies by Qu and colleagues [9, 16, 17] who used a variant of the Luo-Rudy (LR1) ventricular cardiac action potential model [18] to show that manipulating either the kinetics of the L-type calcium current (*I*_*CaL*_) or the activation rate of the delayed rectifier potassium current (*I*_*K*_), can elicit ultra-long action potentials both with and without early after-depolarizations. In some circumstances those action potentials too fail to repolarize. Such cells are said to have ‘bistable’ membrane dynamics because they can reside in either the normal resting state or at an elevated voltage state [15].

We hypothesized that heart cells with bistable membrane dynamics could provide both a substrate and a trigger for arrhythmogenesis. Such cells would have the capacity to initiate fibrillation if they remained depolarized beyond the refractory period of their neighboring cells. The extreme variation in repolarization heterogeneity could also contribute to the breakdown of orderly wave propagation that occurs in fibrillation. In this study we examined electrophysiological recordings of freshly isolated cardiac myocytes for evidence of cells with bistable membrane dynamics. We then undertook a theoretical assessment of how such cells might contribute to the genesis of fibrillation by modeling heart tissue as a sheet of FitzHugh-Nagumo cells [19, 20] using a mixture of monostable and bistable cell types. In particular, we analyzed how the behavior of the bistable cells was influenced by the strength of electrical coupling between cells, the degree of heterogeneity in the tissue, and the presence of anatomical boundaries. Lastly, we confirmed our findings in the same model of the ventricular action potential used by Qu and Chung [16] to study ultra-long action potentials.

## Results

**Fig 1:**
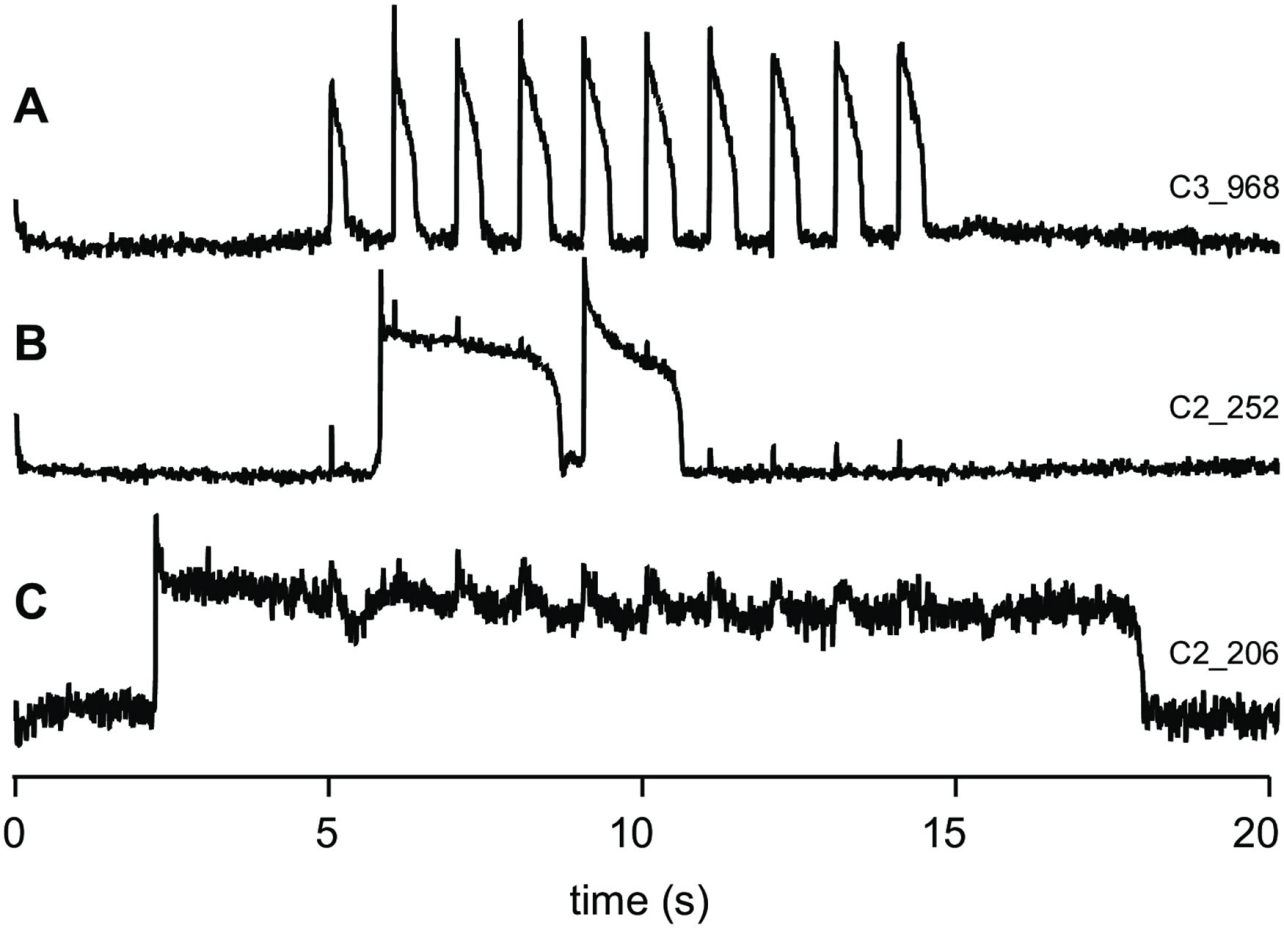
Ultra-long action potentials in cardiomyoctes isolated from rabbit hearts and paced at 1 Hz. (A) Example of a normal train of action potentials. (B) Examples of two ultra-long action potentials that remain depolarized for approximately 2.5 s and 1.5 s respectively. (C) Example of an ultra-long action potential that exceeds 15 s.

Inspired by Kettlewell and colleagues’ [15] observations of bistable action potentials in atrial myocytes, we undertook high throughput optical recordings of ventricular myocytes isolated from rabbit hearts to look for the presence of bistable or ultra-long action potentials. Anticipating that non-repolarising cells would likely become calcium overloaded and die before action potentials could be recorded, the cells were stored in medium containing 0.5 mM Ca^++^, rather than the more commonly used 1.8 mM Ca^++^ (see e.g. [15]). When paced at 1 Hz, 96% of cells (657/685) had action potential durations (APD_90_) in the range 240–880 ms which we classified as normal (e.g. Fig 1A). Very long action potentials (defined as APD_90_ > 1s) were observed in 3.8% of cells (26/685) and ultra-long action potentials (defined as APD_90_ > 2s) were observed in 0.3% of cells (2/685). Selected examples are shown in Fig 1B and 1C respectively.

Bistability—or close to it—is the driving principle behind ultra-long action potentials. Hence the existence of ultra-long action potentials in isolated myocytes suggests that myocytes with bistable action potentials may exist in vivo. One would expect that a small population of such cells would not exert any observable effects on the overall electrical activity in a well-coupled myocardium. However, that may change if disease processes were to reduce the cell-to-cell coupling or to increase the proportion of bistable cells in the tissue. In the next section we use numerical simulations to explore how bistable cells alter the behavior of cardiac tissue.

### Computational model

In order to examine the impact of bistable cells in an electrically coupled myocardium, we modeled the heart as a two-dimensional sheet of excitable cells with generalized FitzHugh-Nagumo [19, 20] dynamics,

$$\begin{array}{cc} \tau_{1}\;\frac{\partial V}{\partial t} & =V-\frac{1}{3}V^{3}-W+c^{2}\;\frac{\partial^{2}V}{\partial x^{2}}+d+I \end{array}$$ (1)

$$\begin{array}{cc} \tau_{2}\;\frac{\partial W}{\partial t} & =V+a-bW \end{array}$$ (2)

where *V*(*x*, *t*) represents the membrane potential of the cell at position *x*∈R^2^. The recovery variable *W*(*x*, *t*) is an abstract representation of the repolarizing currents that return the membrane potential to rest. Parameter *I*(*x*, *t*) is a spatiotemporal stimulus that is applied to the medium to initiate a propagating wave. Parameters *a*, *b* and *d* are constants that dictate the dynamics of the individual cells. Parameter *c*^2^ represents the strength of the electrical coupling between adjacent cells. Eqs (1 and 2) encapsulate the fundamental character of the heart as an excitable medium without the computational burden of excessive anatomical or molecular detail.

**Fig 2:**
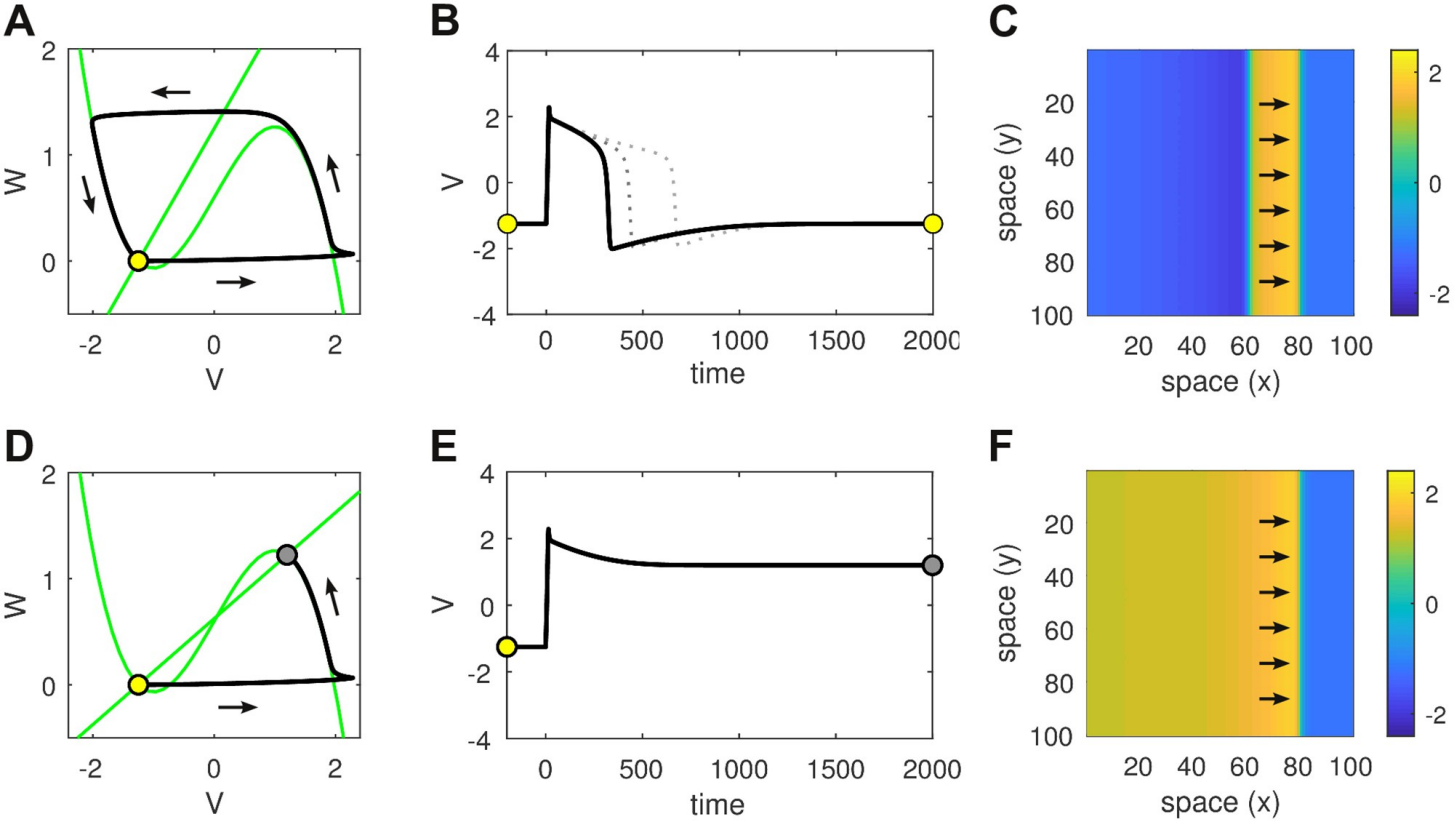
Comparison of monostable versus bistable dynamics in both the isolated cell and the homogeneous spatial medium. **(A)** Phase plane of the isolated cell in the monostable regime (*b* = 1). The nullclines are shown in green. The single fixed point (open circle) is stable. It represents the resting membrane potential. The system can be perturbed from rest by a brief injection current (*I* = 2 for 15 ms) that initiates a trajectory (black line) which makes a large excursion in phase space before returning to rest. That excursion corresponds to the action potential. **(B)** Time plot of the action potential in the monostable regime. Solid line for *b* = 1. Dotted lines for *b* = 1.5 and *b* = 1.75. **(C)** Snapshot of the corresponding traveling wave in a homogeneous sheet of 100 × 100 monostable cells with coupling parameter *c* = 1. Arrows indicate the direction of travel. Color indicates the membrane potential. **(D)** Phase plane of the isolated cell in the bistable regime (*b* = 2). Perturbing the resting state (open circle) induces a transition to the up-state (filled circle). **(E)** Time plot of that transition. **(F)** The corresponding traveling front in the spatial medium.

Excitability is best understood by analyzing the phase plane for an isolated cell (Fig 2A and 2D and Eqs 5 and 6 in Methods). The phase plane describes how the states of *V* and *W* evolve with respect to each other. The nullclines (green; Eqs 7 and 8) indicate those points in the phase plane where the time derivatives of each state variable are zero. Equilibrium points exist where the nullclines intersect. We chose parameters *a* = 1.25 and *d* = 0.6 so that the nullclines intersected near the left-hand knee of the cubic nullcline (*V* = −1.25, *W* = 0). Such a configuration is a known requirement for excitability [21].

Parameter *b* dictates the slope of the linear nullcline which pivots on the equilibrium point for our choice of *a* and *d*. For the case of *b* = 1, the nullclines intersect exactly once and the system is *monostable* (Fig 2A). In this regime, an action potential can be elicited in the resting cell by injecting it with a brief injection current (Fig 2B). The time and space constants (*τ*_1_ = 10, *τ*_2_ = 400, *dx* = 1) were chosen so the shape of the action potential resembled those of cardiac cells. This action potential propagates as a planar wave in a homogeneous medium (Fig 2C). The speed of propagation is dictated by the coupling strength (*c*) and, to a lesser extent, the excitability threshold (*d*).

Small increases in *b* prolong the action potential (dotted lines in Fig 2B) by increasing the decay rate of the recovery variable. In the context of cardiac cells, it is akin to shortening the lifetime of the repolarizing currents. However there is a limit to how much the recovery variable can be shortened before repolarization dramatically fails. Fig 2D shows the case of *b* = 2 where the nullclines intersect at three points in the phase plane. Two of these fixed points are stable (marked by circles) and the other is unstable. The lower stable fixed point (open circle) corresponds to the same resting membrane potential (*V* = −1.25) as before. Whereas the upper stable fixed point (filled circle) represents a newly created high-voltage state (*V* = 1.2) which we refer to as the *up-state*.

Crucially, the resting state and the up-state co-exist for the same choice of parameters, hence the cell is *bistable*. It can be transitioned from the resting state into the up-state using the same brief injection current as before (Fig 2E). In the spatial medium, that transition from resting state to up-state propagates as a traveling front which ultimately recruits the entire medium (Fig 2F).

### Stability analysis

**Fig 3:**
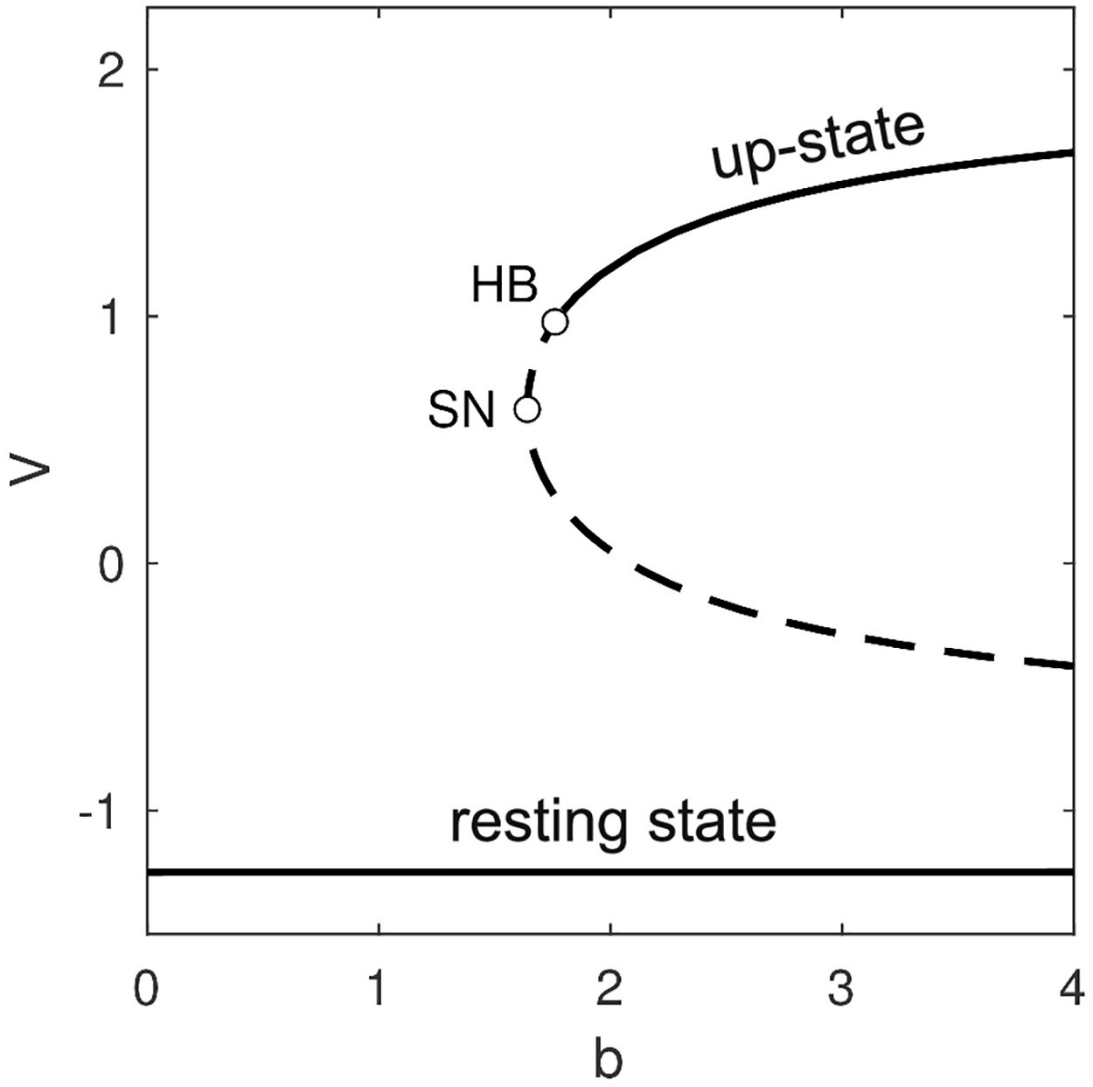
Bifurcations in the steady-states of the single cell. The resting state (*V* = −1.25) is stable (solid line) for all values of parameter *b* > 0. A pair of unstable fixed points (dashed lines) emerge via a saddle-node (SN) bifurcation at *b* = 1.64. The lower branch is the saddle and the upper branch is the node. The node becomes stable via a Hopf bifurcation (HB) at *b* = 1.76. The stable up-state co-exists with the stable resting state for *b* ≥ 1.76.

To better understand the onset of bistability in the isolated cell, we used numerical continuation to follow the steady-state membrane potential over a range of values of *b* (Fig 3A). The technique involves tracking the dynamical stability of a given steady-state solution while slowly changing one or more parameters of the system. The stability of the steady-state is ascertained from the eigenvalues of the linearized system of equations. Those eigenvalues quantify the growth (or decay) of small perturbations to the steady-state. A steady-state is stable only if all perturbations decay over time.

The resting state (*V* = −1.25) was found to be stable (solid line) for all values of *b* that we investigated. Furthermore, it is the only solution that exists for *b*<1.64 meaning that regime is monostable. At *b* = 1.64 an additional pair of unstable fixed points (dashed lines) emerge via a saddle-node (SN) bifurcation. Geometrically, this occurs when the linear nullcline comes into contact with the upper branch of the cubic nullcline. However the newly emerged fixed points are both unstable so the dynamical regime is still monostable at that point. Bistability does not emerge until *b* = 1.76 where the up-state becomes stable via a supercritical Hopf bifurcation (HB).

### Mixed medium of normal and non-repolarizing cells

**Fig 4:**
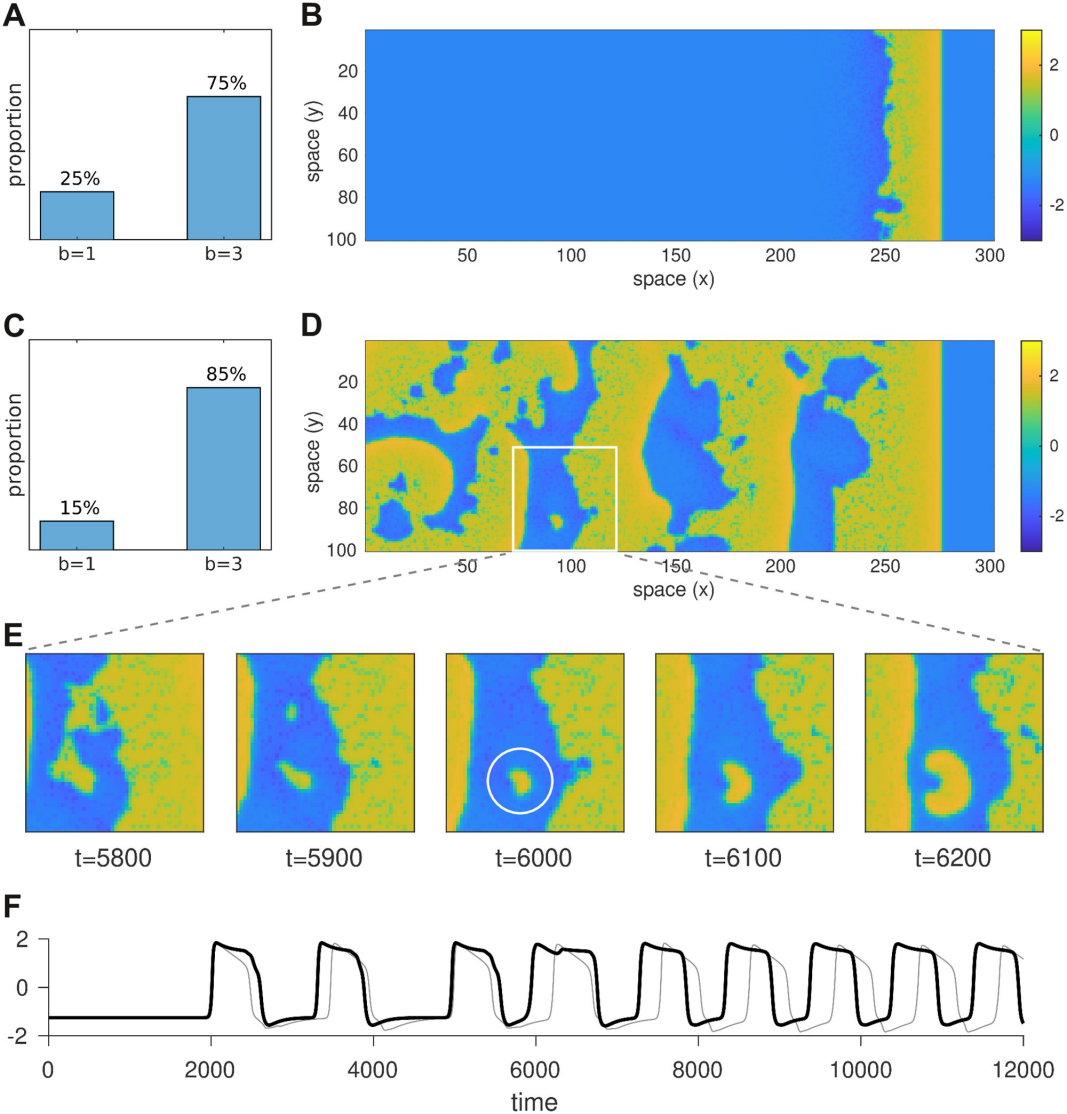
Effect of mixing monostable and bistable cells in the same tissue. Here we compare wave propagation in two simulations of 100 × 300 heterogeneous media where each cell was randomly assigned either *b* = 1 (monostable) or *b* = 3 (bistable) according to the distributions in panels A and C. A rightward traveling wave was initiated in the resting medium by briefly stimulating the cells at the left hand boundary. Snapshots of the respective simulations (*t* = 6000 ms) are shown in panels B and D. The former supports a functional propagating wave whereas the latter leaves self-sustained ectopic activity in its wake. The coupling strength (*c* = 0.6) is identical in both cases. Panel E shows successive snapshots of the 50x50 region marked with a white square. The ectopic activity in that region grew from a small cluster of cells that failed to repolarize (white circle). The time series of the cell at the center of the white circle (*x* = 93, *y* = 87) is shown in panel F (black). The light gray trace in panel F is that of a nearby cell (*x* = 93, *y* = 77). See S1 Video for an animated version of this figure.

To assess how the presence a limited number of bistable cells might impact the macroscopic electrical properties of heterogeneous cardiac tissue, we constructed a 300 × 100 sheet of cells in which the cells were randomly configured to be either monostable (*b* = 1) or bistable (*b* = 3) in varying proportions. In each case the sheet was stimulated briefly (*I* = 2 for 15 ms) at the left-hand boundary to induce a rightward propagating wave (Fig 4A–4D). We found that the heterogeneous medium could tolerate a surprisingly large proportion of bistable cells (75%) and still support a functional traveling wave (Fig 4A and 4B). When that proportion was increased to 85%, the propagating wave initiated persistent disordered spatiotemporal activity that resembles fibrillation (Fig 4C and 4D). It emerged from a small cluster of bistable cells that failed to repolarize in the wake of the traveling wave (Fig 4E). Those ‘rogue’ cells were thus able to re-excite their neighbors once they had recovered from refractoriness. The ensuing activity was disordered because the heterogeneous arrangement of monostable and bistable cells breaks the symmetry of the system. Symmetry is further broken by the ability of bistable cells to dwell arbitrarily in either the resting state or the up-state. Fig 4F illustrates a bistable cell (black) dwelling in the up-state for variable periods compared to the regular duty cycle (light gray) of a nearby monostable cell. Interestingly, the bistable cell does not remain in the up-state indefinitely as it would in the homogeneous medium. Instead, it is aperiodically switched between the resting state and the up-state due to the influence of normal cells in the neighborhood. Eventually that switching settles into a regular rhythm as the global activity converges to a stable spatiotemporal pattern. Arrhythmias can thus emerge spontaneously from the interaction between normal cells and bistable cells.

### Reduced model

**Fig 5:**
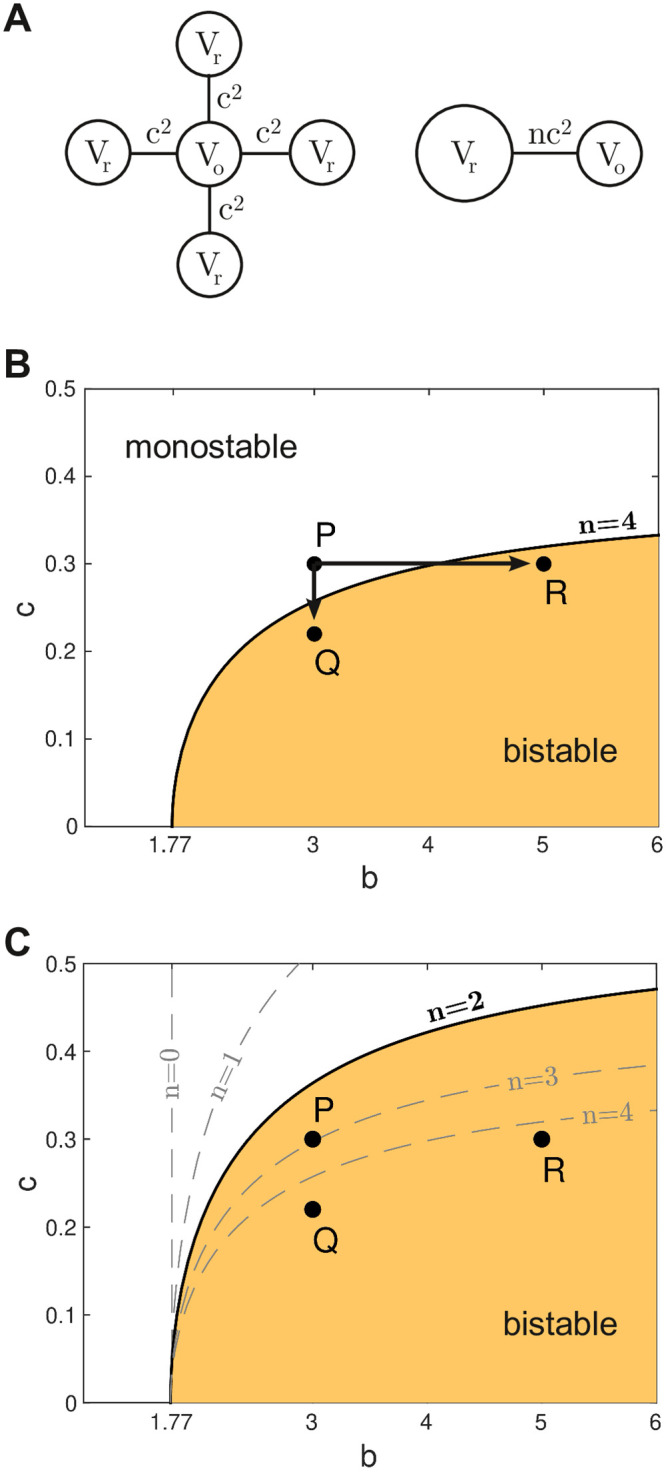
Stability analysis of the reduced model. (A) Schematic of the model. Left: A single bistable cell (*V*_*o*_) is coupled to *n* identical cells which are all clamped at rest (*V*_*r*_). The coupling strength is *c*^2^. Right: The resting cells are lumped together to form the reduced model where the equivalent coupling is *nc*^2^. (B) Stability map for the reduced model with *n* = 4 neighbors. The shaded region indicates those configurations of *b* and *c* where the cell operates in the bistable regime. Points *P* = {3, 0.3}, *Q* = {3, 0.22} and *R* = {5, 0.3} are described in the text. (C) Stability map for the case of *n* = 2 neighbors. The stability boundaries for *n* = 0, *n* = 1, *n* = 3 and *n* = 4 neighbors are included for comparison.

To further analyze the conditions under which a bistable cell fails to repolarize when it is embedded in a medium, we constructed a reduced version of our model where a single cell of interest interacts with a hypothetical resting medium (Fig 5A, left). The membrane potential of that cell (*V*_*o*_) was free to vary while all other cells were fixed at the resting potential (*V*_*r*_ = −1.25) on the assumption that their membrane potentials were dominated by the medium acting as a global syncitium. Under these assumptions, the gap current flowing into the cell of interest from one neighboring cell is *I* = *c*^2^(*V*_*r*_ − *V*_*o*_). Since the neighboring cells were all identical, their contributions were lumped together as one equivalent cell (Fig 5A, right). The combined gap current being *I* = *nc*^2^(*V*_*r*_ − *V*_*o*_) where *n* is the number of neighbors.

The equations for the reduced model were thus defined as,

$$\begin{array}{cc} \tau_{1}\;\frac{\partial V_{o}}{\partial t} & =V_{o}-\frac{1}{3}V_{o}^{3}-W_{o}+d+nc^{2}\left(V_{r}-V_{o}\right) \end{array}$$ (3)

$$\begin{array}{cc} \tau_{2}\;\frac{\partial W_{o}}{\partial t} & =V_{o}+a-bW_{o} \end{array}$$ (4)

where the parameters are the same as for the single cell equations. Indeed, Eqs (3 and 4) have the same basic form as the single cell Eqs (5 and 6) hence the bifurcation structure is the same as Fig 3 except that here the location of the Hopf bifurcation (HB) depends on *n* and *c* as well as *b*. We mapped out this critical relationship for the case of *n* = 4 by numerically following the Hopf point while allowing *b* and *c* to vary as free parameters. The resulting curve (Fig 5B) describes the stability boundary between the monostable and bistable operating regimes for all configurations of the reduced model. The shaded region indicates those parameter configurations where the cell of interest supports both a stable resting state and a stable up-state, despite the repolarizing influence of the resting medium in which it is embedded. Conversely, the unshaded region indicates those parameter configurations where the cell of interest behaves in a monostable fashion. Cells with those configurations are guaranteed to repolarize when they are embedded in a resting medium even though those with *b* > 1.77 would not do so in isolation.

### Progression of disease

The stability analysis of the reduced model suggests three pathways by which a cell may transition from normal (monostable) behavior—where the cell always repolarizes—to abnormal (bistable) behavior where the cell fails to repolarize. The first pathway involves increasing the intrinsic bistability characteristics of the cell while holding the coupling strength fixed. It is illustrated in Fig 5B by moving the parameter configuration from the monostable regime at *P* to the bistable regime at *R*. In this case, the stability boundary is crossed at *b* ≈ 4. We envisage this pathway as corresponding to some underlying change in the cell’s physiology that impairs its repolarizing currents.

The second pathway involves an overall reduction in the coupling strength between cells while all other aspects of the cell physiology remain unchanged. It is illustrated in Fig 5B by moving from configuration *P* to configuration *Q*. Here the stability boundary is crossed at *c* ≈ 0.26. We envisage this pathway as representing an overall reduction in the conductance of the gap junctions.

The third pathway involves a reduction in the number of neighboring cells. It is illustrated in Fig 5C by the shift in the stability boundary when the number of neighbors is reduced from *n* = 4 to *n* = 2 thus transforming configuration *P* from monostable to bistable. We interpret this particular pathway as highlighting a natural deficit that is faced by cells on tissue boundaries rather than a progressive loss of connectivity over time. It illustrates how cells on tissue boundaries are more susceptible to abnormal behavior than their counterparts in the midfield of the tissue. These boundary effects may explain why the pulmonary vein often appears to be the source of ectopic activity in clinical observations.

### Bistability as a driver of ectopic activity in tissue

**Fig 6:**
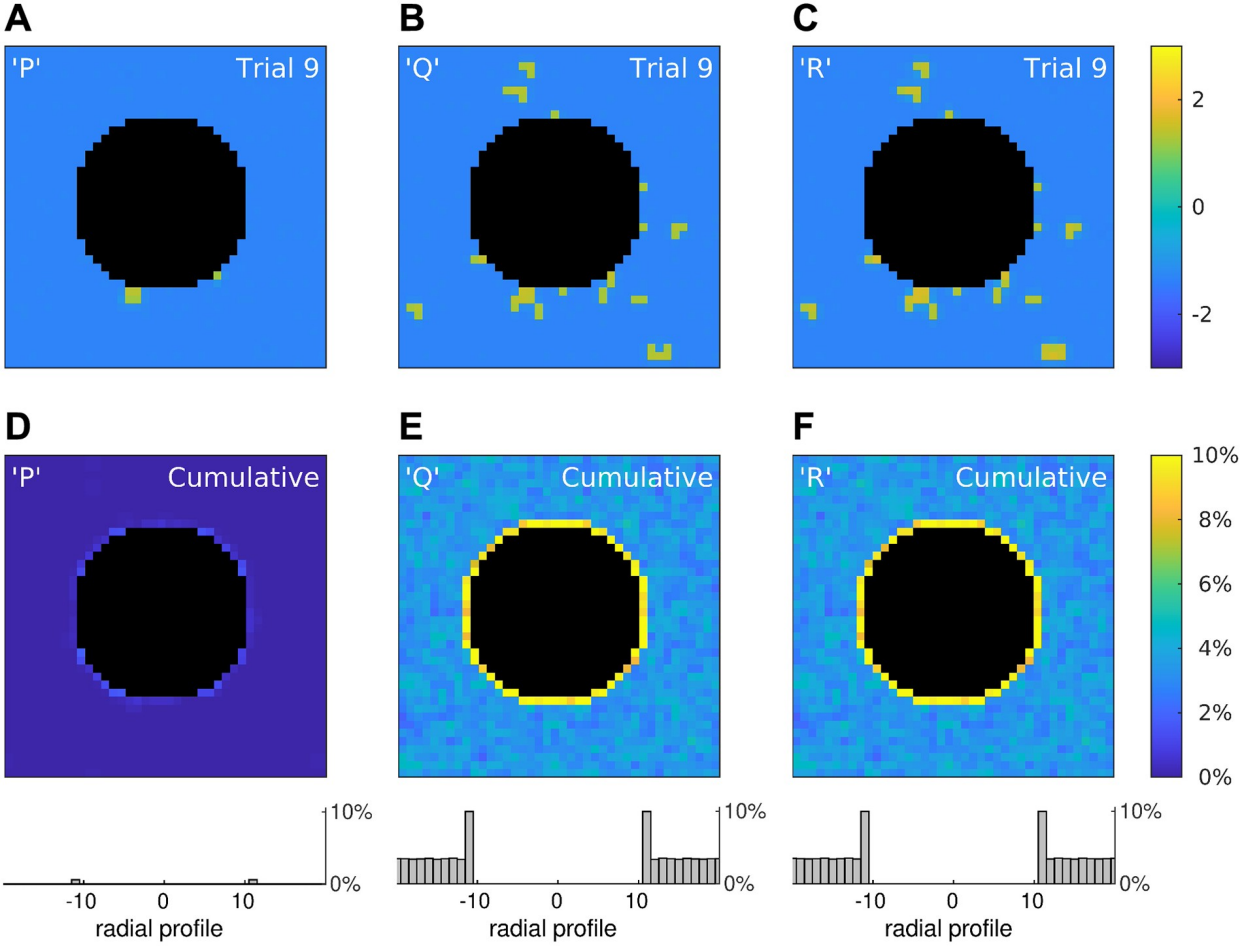
Rogue activity emerges preferentially at tissue boundaries. (A–C) Snapshots of three simulations of a 40 × 40 medium in which 10% of the cells were configured to points *P*, *Q* or *R* in the stability map of the reduced model (Fig 5). The snapshots were taken at *t* = 1000 ms post-onset of a spatially uniform stimulus. The central annulus (black) represents a tissue boundary where cells are absent. The edges of the simulation domain used periodic boundary conditions to eliminate artificial boundary effects. The color scale indicates the membrane potential of the cells. (D–F) Cumulative results for 1000 trials where color is the percentage of cells that remained depolarized in the long term. Histograms (bottom) show the averaged radial profiles. See S2 Video for an animated version of this figure.

Our analysis of the reduced model predicted that a bistable cell in a hypothetical resting media will behave abnormally when the coupling strength is reduced or when it is coupled to fewer neighbors. We tested these predictions in the full model by configuring 10% of the cells in the medium to be intrinsically bistable and the remaining 90% to be intrinsically monostable. We reasoned that this sparse allocation of bistable cells would closely match the reduced model where all neighboring cells were assumed to be at rest. We used a small 40 × 40 spatial domain so that individual cells could be visualized easily. We also introduced an annulus into the medium to test the prediction that ectopy is more likely to arise at tissue boundaries. The annulus represents a vein or artery in the wall of the heart and was modeled as a region with zero coupling between cells. Cells on the boundary of the annulus had either *n* = 2 or *n* = 3 neighbors depending on the spatial discretization of the local curvature (Fig 6). The spatial domain itself had periodic boundary conditions to eliminate artificial boundary effects.

We simulated the full model under three conditions where the configurations of the bistable cells were taken from points *P*, *Q*, *R* on the stability map for the reduced model, respectively. Configurations *Q* and *R* were both predicted to be bistable for *n* = 4 and *n* = 2 neighbors, so those cells were expected to fail to repolarize no matter where they were located in the tissue. Whereas configuration *P* was predicted to be monostable for *n* = 4 neighbors and bistable for *n* = 2 neighbors. Hence those cells were expected to repolarize normally in the midfield of the tissue but not at the boundary of the annulus where the cells have two neighbors. Configuration *P* also straddles the stability boundary for *n* = 3 where bistability is marginal. So boundary cells with three neighbors were expected to repolarize normally.

For each configuration, the medium was probed for abnormal repolarization by briefly stimulating it with a spatially-uniform stimulus (*I* = 2 for 15 ms) and then observing whether any cells remained depolarized at 1000 ms post-onset of the stimulus. The outcomes of a single trial for each configuration under identical spatial conditions are shown in Fig 6A–6C. A survey of 1000 such trials with random spatial conditions is provided in S2 Video. Overall, those trials confirm that cells with configurations *Q* and *R* can fail to repolarize at any location, whereas those with configuration *P* are most likely to fail when located at the tissue boundary.

Those findings are quantified by the trial-averages (Fig 6D–6F) where color indicates the percentage of trials in which each cell failed to repolarize (*V*>0 at *t* = 1000). The histograms (bottom) are the averaged radial profiles of all trials in each condition. They reveal elevated failure rates for cells on the tissue boundary compared to cells in the midfield. In particular, cells on the boundary for configuration *P* failed to repolarize in 0.6% of trials (SE 0.03) while those in the midfield always repolarized. Whereas cells on the boundary for configuration *Q* failed to repolarize in 10.0% of trials (SE 0.12) and those in the midfield failed in 3.4% of trials (SE 0.03). Similarly for configuration *R* where cells on the boundary failed to repolarize in 10.0% of trials (SE 0.12) and those in the midfield failed in 3.5% of trials (SE 0.03). Indeed, the radial profiles in Fig 6E and 6F are virtually identical because configurations *Q* and *R* are equivalent distances from the stability boundary in Fig 5B.

Normalizing the observed failure rates by the density of abnormal cells in the simulated medium (10%) gives adjusted failure rates of 100% for *Q* and *R* cells on the tissue boundary and 35% in the midfield. The adjusted failure rate for *P* cells on the tissue boundary is 6%, although that figure is likely to be an underestimate because many boundary cells had three neighbors instead of two and so were more likely to repolarize. The cells that did fail were predominantly located at the diagonal quadrants of the annulus, as can be seen in Fig 6D. Overall, the simulations in the full model were consistent the predictions of the reduced model.

### Bistability in the context of physiological variability

**Fig 7:**
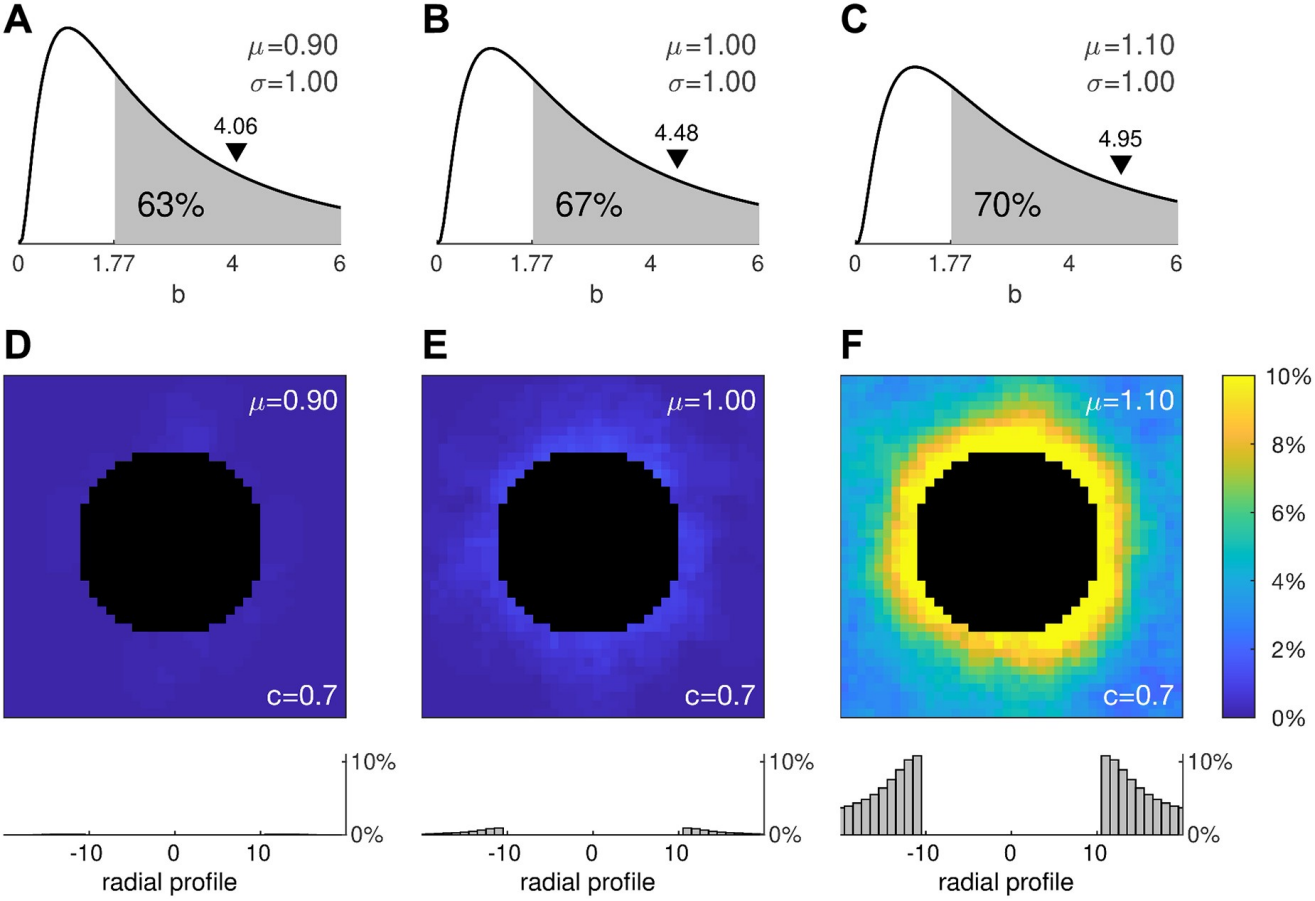
Effect of physiologically realistic distributions of cell heterogeneity. (A–C) Log-normal (*μ*, *σ*) distributions for *μ* = 0.9, *μ* = 1.0 and *μ* = 1.1 where the second moment is fixed at *σ* = 1. The shaded region denotes the area under the curve where *b*>1.77 exceeds the critical value for bistability in the single cell. The black triangle marks the arithmetic mean of the distribution. (D–F) Cumulative results for 1000 stimulation trials using the same protocol as Fig 6. Histograms (bottom) show the averaged radial profiles.

The analysis above considers a simple binary population of cells that that are either intrinsically monostable (*b* = 1) or bistable (*b* = 3). While this approach is appropriate for a theoretical analysis of how abnormal cells with bistable characteristics lead to emergent ectopy in coupled tissue, the physiological reality is that cellular properties are not distributed in this manner. Rather, repolarization times of cardiac myocytes are smoothly distributed [13, 14], extending to non-repolarizing cells at the extreme long tail of the continuum. To approximate this in our simulations we replaced the sparse binomial distribution used in Fig 6 with a log-normal distribution (*μ*, *σ*) as shown in Fig 7. The log-normal distribution has a suitably long tail and satisfies *b* > 0 which prevents runaway growth of the recovery variable (Eq 2).

We reasoned that broadening the log-normal distribution would have a similar effect to that of increasing the proportion of bistable cells in the previous simulations. We tested this prediction by manipulating *μ* while holding *σ* = 1 fixed as shown in Fig 7A–7C. The shaded regions indicate the proportions of cells in each distribution that are intrinsically bistable (*b*>1.77). Following the same protocol as before, we applied a spatially uniform stimulus to a 40 × 40 medium with a central annulus and quantified how many cells remained depolarized at 1000 ms post-stimulus onset. After running a few pilot trials, we fixed the coupling strength at *c* = 0.7.

The trial averages of 1000 simulations in each condition are shown in Fig 7D–7F. The first thing that is evident from these simulations is that the tissue can still repolarize normally despite 63% of its cells being bistable. Failure of repolarization does not emerge until that proportion approaches 67% and it is abundantly evident once that proportion reaches 70%. This remarkable tolerance to the presence of abnormal cells is reminiscent of our earlier observations in the densely mixed medium of monostable and bistable cells (Fig 4).

The second thing that is evident is that ectopic activity emerges first at the tissue boundary. Once again, this is consistent with the reduced model, albeit the ectopy emerges at a higher coupling strength than predicted. That is because the densely populated medium allows more cells to reside in the up-state simultaneously and those cells exert no repolarizing influence on one another. Stronger coupling compensates for that loss by amplifying the contributions from the cells that do repolarize.

**Fig 8:**
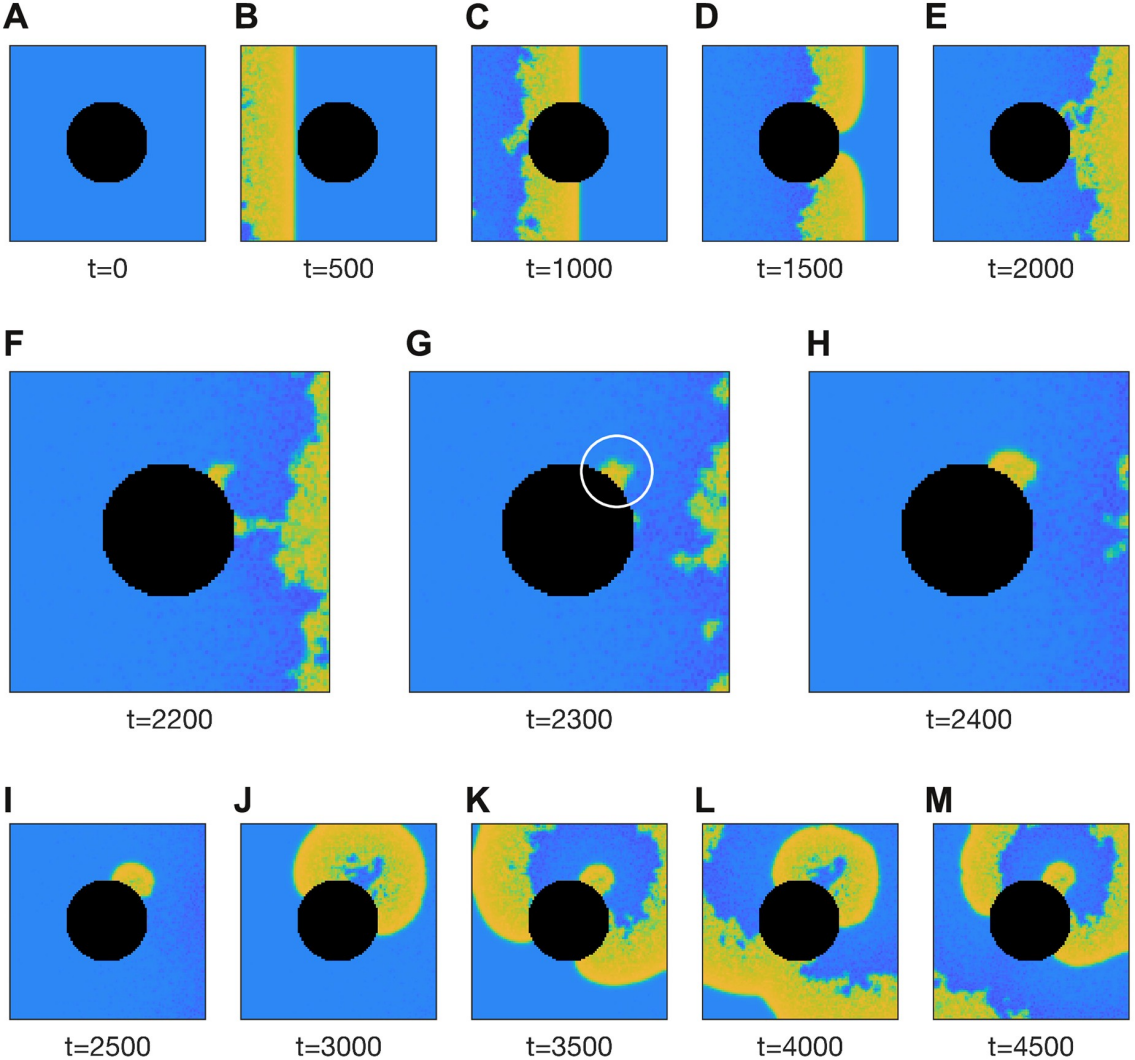
Ectopic activity induced at the tissue boundary by a passing wave. The 100 × 100 medium is briefly stimulated at the left-hand boundary and the ensuing wave propagates to the right. The black region represents the tissue boundary. The white circle in panel G marks the genesis of ectopic activity by cells on the tissue boundary that fail to repolarize. The medium is densely heterogeneous with parameter *b* of each cell drawn randomly from a log-normal distribution (*μ* = 1, *σ* = 1). The coupling coefficient is *c* = 0.7. See S3 Video for an animated version of this figure.

We next examined how that behavior would manifest in the context of a wave of action potentials propagating across densely heterogeneous tissue containing a natural tissue boundary (Fig 8). In this simulation, the medium was configured with the same parameters (*μ* = 1, *σ* = 1, *c* = 0.7) as per Fig 7E. A rightward traveling wave was initiated in the medium by briefly stimulating the cells on the left-hand side. That wave propagated around the annulus and exited the medium to the right (Fig 8A–7E). All cells repolarized normally in the wake of the wave except for a few located on the boundary of the annulus, marked by the white circle in Fig 8G. Those cells subsequently emitted trains of ectopic waves into the surrounding tissue (Fig 8I–7M). The simulation is best viewed in S3 Video. It demonstrates how sustained fibrillation can be initiated by a small number of cells that fail to repolarize in the wake of a normal propagating wave. It also illustrates how cells at tissue boundaries are more susceptible.

### Progression of arrhythmic disease

**Fig 9:**
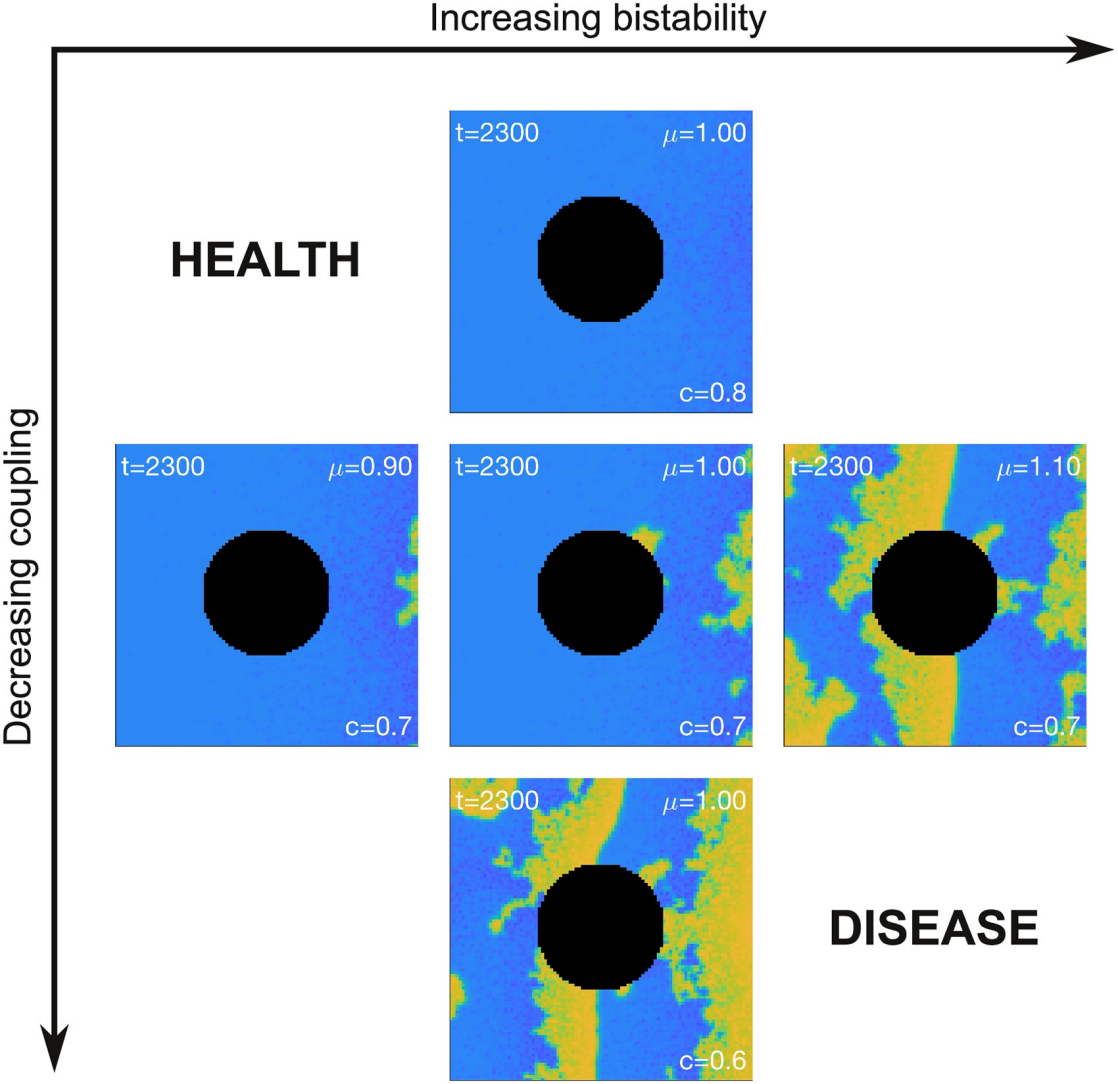
Pathways for the progression of disease. Healthy tissue corresponds to cells with low intrinsic bistability and high coupling (top-left). Disease can progress by increasing bistability or decreasing coupling strength or both. See S4 Video for an animated version of this figure.

The observations above provide a framework for understanding how the presence of cells with bistable electrophysiological characteristics may lead to the emergence and progression of arrhythmic disease in the heart. The relationship between the the prevalence of bistability, the degree of inter-cellular electrical coupling, the presence of a tissue boundary and the emergence of disordered tissue level electrophysiology is summarized in Fig 9. In this scheme, the axes represent the pathways for the progression of disease by increasing bistability (left-to-right) and decreasing inter-cellular coupling (top-to-bottom). Each panel is a snapshot of the simulation using the same protocol as Fig 8. The increase in bistability was achieved by increasing the first moment of the log-normal distribution of *b* from *μ* = 0.9 to *μ* = 1.1, exactly as in Fig 7A–7C. The inter-cellular coupling was decreased from *c* = 0.8 to *c* = 0.6. The impact of the tissue boundary was encapsulated by the annulus. Overall, the transition from healthy electrical activity (top left) to arrhythmic electrophysiology (bottom right) can be achieved by increasing the prevalence of bistable cells or by decreasing the coupling between cells, or both.

### Verification in a biophysical model

**Fig 10:**
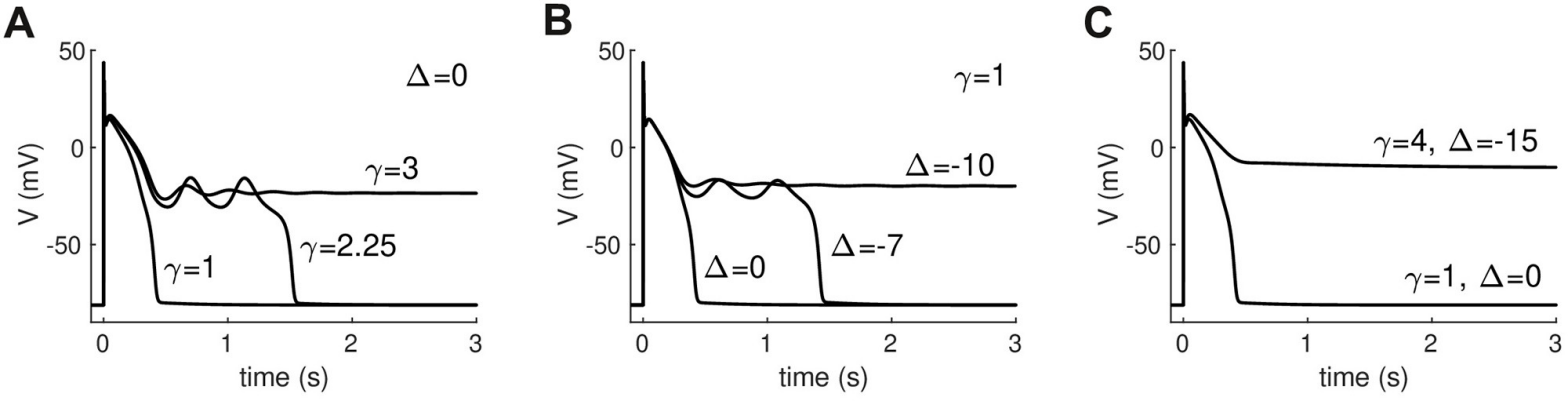
Ultra-long and bistable action potentials in the Qu-Chung [16] model. (A) Repolarization failure induced by slowing the activation rate (1/*γ*) of the delayed rectifier potassium current. (B) Repolarization failure induced by negatively shifting the voltage-dependence of L-type calcium current inactivation by Δ mV. (C) Monostable and bistable action potential configurations used in subsequent simulations. The values *γ* = 4 and Δ = −15 were chosen to produce a robust bistable action potential that does not repolarize in the isolated cell. All other parameters are indentical to [16] except for *I*_*K*1,*max*_ = 0.3.

While the FitzHugh-Nagumo model [19, 20] is a tractable choice for analysis, it is not a biophysical membrane model. In particular, it lacks important features of cardiac cells such as conduction velocity restitution. We therefore sought to verify our findings in the biophysical model used by Qu and Chung [16] to study ultra-long action potentials. It is a variant of the Luo-Rudy (LR1) model of the ventricular action potential [18] in which the voltage window of the L-type calcium current (*I*_*CaL*_) and the activation speed of the delayed rectifier potassium current (*I*_*K*_) can both be manipulated. Ultra-long and bistable action potentials arise in this model when either the activation speed of *I*_*K*_ is slowed by increasing *γ* (Fig 10A) or when the activation window of *I*_*CaL*_ is shifted to a lower voltage range by decreasing Δ (Fig 10B) or both.

We constructed a 20 × 60 sheet of cells and repeated the same scenario as Fig 4 where a randomly selected subset of the cells were configured to be strongly bistable (*γ* = 4, Δ = −15) while the remaining cells were monostable (*γ* = 1, Δ = 0). The profiles of the action potentials in the single cell are shown in Fig 10C. All parameters are identical to Qu and Chung’s model [16] except for *I*_*K*1,*max*_ which we reduced from 0.605 to 0.3 to lower the firing threshold of the cell and promote wave propagation.

**Fig 11:**
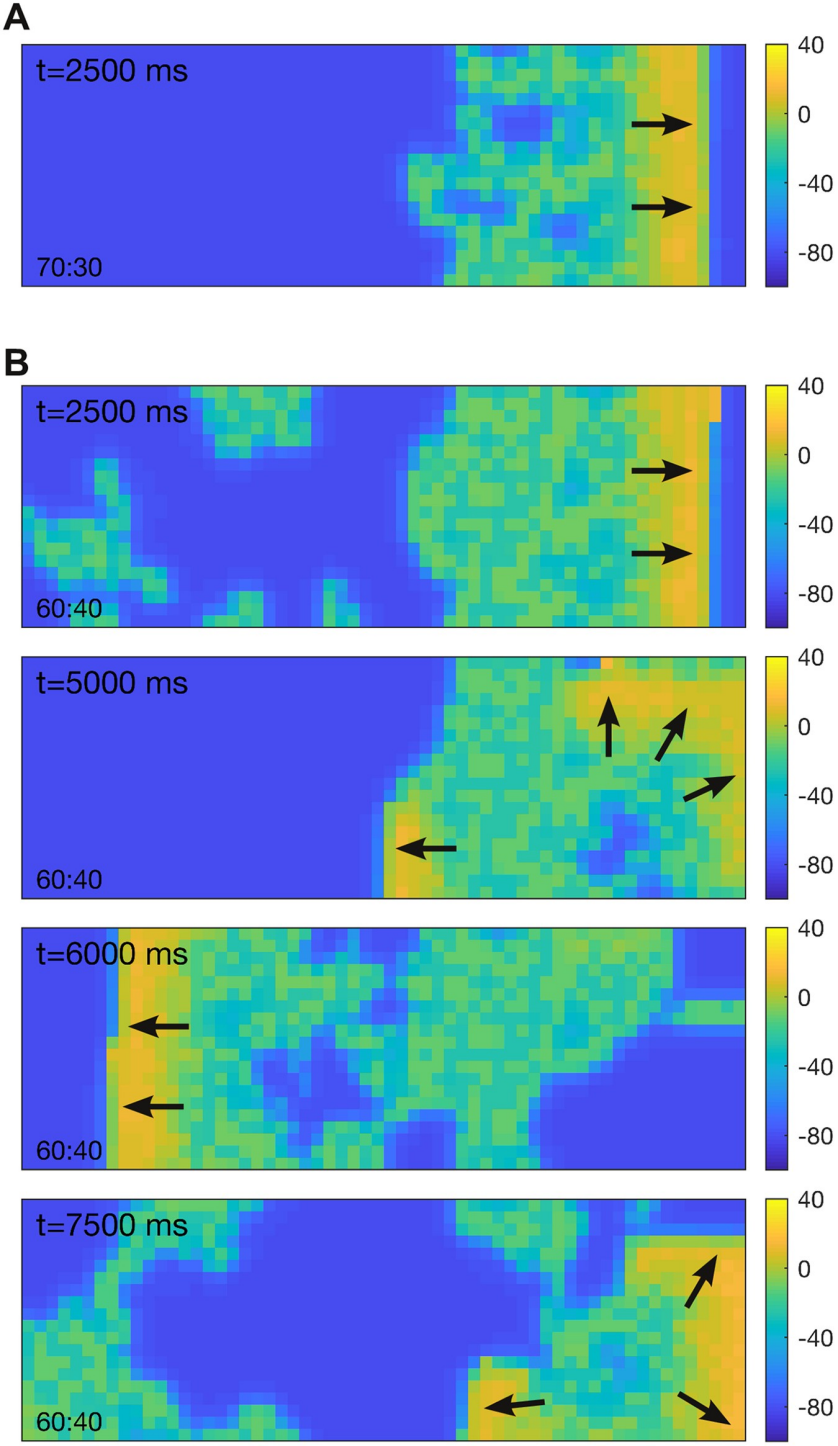
Effect of mixing monostable and bistable cells in a biophysical model. The medium comprises 20 × 60 ventricular cells with action potentials described by the Qu-Chung model [16]. Cells on the left-hand boundary were stimulated briefly (80 pA for 0.5 ms) to induce a rightward traveling wave. Color indicates the membrane potential of the cells. Arrows indicate the direction of propagation. (A) Case of a medium with 70% monostable cells (*γ* = 1 and Δ = 0 mV) and 30% bistable cells (*γ* = 4 and Δ = −15 mV). (B) Case of 60% monostable and 40% bistable cells. The coupling strength (*c* = 0.15) is identical in both cases. Neumann (no-flux) conditions were applied at the boundaries. See S5 Video for an animated version of this figure.

Fig 11A shows a snapshot (*t* = 2500 ms) of the spatial model with 70% monostable and 30% bistable cells. In that case, the wave (yellow) propagates normally from left to right despite the presence of bistable cells. Some of those cells dwell in the depolarized state (green) for a while but they do repolarize in the long term, albeit some faster than others. Fig 11B shows the case where the medium was configured with 60% monostable and 40% bistable cells. The population of bistable cells in this simulation is a superset of that used in panel A. In this case, the wave still propagates from left to right but it leaves a large proportion of depolarized cells in its wake. As anticipated, those depolarized cells triggered new ectopic waves of activity in their resting neighbors. That activity appears to be self sustained, at least for the 10 s duration of the simulation. See S5 Video.

The behavior of the biophysical model differs somewhat from that of the FitzHugh-Nagumo model in that the depolarized state does not correspond to the peak of the action potential. Consequently, cells in the depolarized state can both block the conduction of incoming waves as well as initiate outgoing waves in adjacent cells. Nevertheless, these simulations demonstrate that bistable action potentials can initiate arrhythmias in a biophysically realistic cell model.

## Discussion

Recent studies suggest that cardiac myocytes could, under some circumstances, fail to repolarize on their own accord because of inherent bistability in their membrane dynamics [15]. To explore the implications of this phenomenon we modeled cardiac tissue as a simple excitable medium [19, 20] containing a mixture of cells with either monostable or bistable membrane dynamics. We found that the medium could tolerate a surprisingly large number of bistable cells and still operate normally because the monostable cells impelled the bistable cells to repolarize. Nonetheless there was an upper limit to how many bistable cells the medium could accommodate before normal function was abruptly lost. At that point, the orderly propagation of cardiac waves gave way to disorderly spatiotemporal activity that resembled cardiac fibrillation. Analysis revealed that the propensity to develop fibrillatory activity was determined by a three-way relationship between the degree of bistability within the cells, the strength of coupling between the cells, and the number of adjoining cells. Gradual changes in each of these factors, as might occur with the progression of cardiac disease, will lead to a tipping point in the dynamical behavior consistent with a sudden onset of cardiac arrhythmias in patients with well-established heart disease [2, 22].

### Bistability of cardiomyocyte membrane dynamics

In standard physiological preparations, isolated cells that do not repolarize would normally die from the toxicity of calcium overload. Nonetheless, bistable membrane dynamics have been observed in isolated cardiac myocytes under controlled experimental conditions. For example, Kettlewell and colleagues [15] observed them in patch clamped atrial myocytes where the endogenous calcium membrane current was blocked by nifedipine and replaced by a dynamic clamp protocol. Our own observations of ultra-long action potentials in ventricular myocytes adds support for the existence of cells with bistable membrane dynamics. In our case, the myocytes were perfused with unusually low levels of calcium to avoid toxicity. Whether such cells could survive *in vivo* remains an open question. Our numerical simulations suggest that bistable cells might avoid calcium toxicity if they are routinely repolarized by the electrotonic influence of normal cells in the vicinity. Hence cells that fail to repolarize of their own accord might still survive in predominantly normal heart tissue.

### Factors that influence the onset of arrhythmogenesis

Unlike bistable action potentials, ultra-long action potentials do eventually repolarize but not necessarily before eliciting ectopy. The dynamical theory of EADs by Qu and Chung [16] suggests that ultra-long action potentials correspond to a quasi-equilibrium state at the plateau voltage and that EADs occur when the plateau voltage loses stability through a Hopf bifurcation—evident by a growing oscillation in the membrane potential. The presence of EADs is often regarded as a surrogate marker for pro-arrhythmic behavior.

In our case, the pro-arrhythmic mechanism is not due to the presence of EADs but is instead due to cells that remain in the depolarized state. In the case of a homogeneous bistable medium, the depolarized state recruits the entire domain and never returns to rest. However the behavior is very different for a heterogeneous medium where the bistable cells are repeatedly driven between the up-state and the resting-state by the action of their neighboring cells. We considered three mechanisms by which bistable cells in a heterogeneous medium can induce fibrillatory activity. Namely (i) increasing the degree of bistability in the cell’s membrane potential, (ii) decreasing the strength of coupling between cells, and (iii) reducing the number of neighboring cells to which the bistable cell is electrotonically coupled. We describe each of these three mechanisms in the following sections.

#### Increasing bistability

Bistability in the generalized Fitzhugh-Nagumo Eqs (1 and 2) is governed by the rate at which the recovery variable decays to rest, represented by parameter *b*. Increasing *b* shortens the time window where the recovery variable can actively repolarize the membrane potential. This prolongs the plateau of the action potential until bistability abruptly emerges at the critical value of *b* = 1.77. At that point, the cell is no longer capable of repolarizing on its own. Nonetheless, it can still be impelled to repolarize by its neighboring cells which drain it of residual current. In doing so, the up-state of the bistable cell is destabilized and the membrane returns to rest under its own dynamics. Even so, this can only happen when the decay rate of the recovery variable is not too large. Otherwise the up-state becomes too strong for the draining currents to overcome. In that case, the cell remains permanently depolarized. The tables are now turned as the ‘rogue’ cell drives current back into the neighboring cells, causing them to depolarize again and again. The cell thus emits spontaneous waves of action potentials into the surrounding tissue which in turn degenerate into fibrillating activity patterns. This path to arrhythmogenesis corresponds to moving from point *P* to point *R* on the stability map (Fig 5B).

#### Decreasing coupling strength

The second mechanism of arrhythmogenesis involves a decrease in coupling strength. The role of reduced gap junction coupling in promoting arrhythmogenesis is very well documented in experimental systems [23, 24]. A reduction in cell-to-cell coupling diminishes the electrotonic currents that couple cells so relives the dampening effect of neighbouring tissue in limiting the emergence of ectopy (i.e. reduces the so-called sink-source mismatch [25, 26]). In our modeling studies, the up-state of the bistable cells gain stability as the neighboring cells lose their influence. Eventually they become permanently depolarized and subsequently emit spontaneous waves into the surrounding tissue. This path to arrhythmogenesis corresponds to moving from point *P* to point *Q* on the stability map (Fig 4B). The arrhythmic behavior manifests first in those cells with the most extreme expressions of bistability—those with the highest *b*. Subsequent reductions in the coupling strength sees the recruitment of those cells with lower expressions of bistability. A reduction in coupling strength is concomitant with a reduction in wave propagation speed. Hence wave speed could serve as a biomarker to distinguish path *P* → *Q* from path *P* → *R* in physiological observations.

#### Reducing the number of connections

The third mechanism promoting arrhythmogenesis involves a reduction in the number of neighbors to which the bistable cell is coupled. In this scenario, the intrinsic bistability of the cell and the conductance of the cell-to-cell coupling are unchanged. The bistable cell merely has fewer neighbors to impel it to repolarize. This path to arrhythmogenesis corresponds to moving the boundary of the stability map (Fig 5C). It might arise through progressive tissue death, or through the intrusion of fibrosis into healthy tissue. Reduced connectivity also arises naturally at tissue boundaries where cells have fewer neighbors than their counterparts in the midfield of the tissue. Furthermore, our simulations show that ectopic activity is prone to manifest first at tissue boundaries, irrespective of whether it is driven by path *P* → *Q* or *P* → *R*. This boundary effect may explain why atrial fibrillation typically originates from the anatomical site where the pulmonary vein attaches to the wall of the heart [27]. Previous studies have considered that such tissue boundaries may facilitate ectopic triggers by reducing the electrotonic loading on the effected cell [28]. Our proposal differs in that it does not rely on an external trigger as a precursor. Instead, ectopy emerges intrinsically from a cell that fails to repolarize following a normal heartbeat.

In that sense, our proposal bears more similarity to the ‘R-from-T’ mechanism of arrhythmogenesis in Long QT syndrome [17] whereby ectopy emerges from contiguous regions of cells with prolonged action potentials in the wake of a normal heartbeat. In that case, the prolonged region simultaneously contributes to the genesis of EADs and the topological conditions for reentry. The trigger and the vulnerable substrate are thus combined into the one mechanism [17, 29]. Interestingly, the geometric shape of the prolonged region can alter its ability to emit an ectopic beat [10]. Paradoxially, shapes with acute angles are more vulnerable than regions of low curvature, in apparent contradiction to the source-sink hypothesis [25]. In our case, ectopy can emerge from randomly distributed cells without the need for contiguous heterogeneities.

### Impact of disease progression

The most clinically important cardiac arrhythmias, atrial fibrillation and ventricular fibrillation, are invariably associated with significant underlying heart disease [1, 2]. One of the major difficulties with managing these arrhythmias is that despite often decades of progressive heart disease these arrhythmias have an abrupt onset that cannot be predicted in advance [2, 22]. The predictions from our modeling of bistable membrane dynamics indicates that this is exactly what one would expect, i.e. the gradual deterioration in cell connectivity, reduction in number of neighbors due to fibrosis, and or increasing extent of bistability can all combine to lead to a sudden tipping point. Our model, however, cannot reproduce the intermittent nature of paroxysmal atrial fibrillation or explain why some arrhythmias spontaneously terminate. This may be due to the lack of action potential duration restitution in the FitzHugh-Nagumo model [30]. We did observe spontaneous termination of arrhythmias in some runs of our biophysical model however it was never clear whether those terminations were due to the membrane dynamics or due to boundary effects in our small spatial domain (20 × 60). Future studies will allow further investigation of the physiological mechanisms underlying bistable membrane dynamics. Nonetheless, the causes of fibrillation are multifactorial and different mechanisms are likely to predominate in different patient populations [31]. Bistable action potentials are but one possible cause.

## Methods

### Ethics statement

This project was approved by the Royal North Shore Hospital Animal Care and Ethics Committee. The Committee operates in accordance with the New South Wales Animal Research Act (1985), Animal Research Regulation (2010) and the Australian code for the care and use of animals for scientific purposes (8th ed. 2013). All studies were performed on isolated cells with no experimental procedures performed on living animals.

### Electrophysiology

Cardiac myocytes were isolated from New Zealand White rabbits using a collagenase digestion protocol as previously described [32, 33]. After isolation, cells were stored in Tyrodes solution containing (in mM) 140 NaCl, 5.6 KCl, 0.5 CaC12, 0.44 NaH2P04, 10 glucose, 1.0 MgC12, and 10 N-2-hydroxyethylpiperazine-N’-2-ethanesulfonic acid (HEPES) and the pH titrated to 7.40 with 1 M NaOH. Cells were used 2-6 hours after isolation.

Isolated myocytes were loaded with the Fluovolt voltage-sensitive fluorescence dye as described in the suppliers manual (Thermo Fisher Scientific, Waltham, MA, USA). Optical measurement of action potentials were recorded on a Kinetic imaging cytometer (KIC IC-200, Vala Sciences, San Diego, CA, USA). Cells were stimulated at 1 Hz and images acquired at 100 Hz using CyteSeer Scanner v2.2.32.0 software (Vala Sciences, San Diego, CA, USA). The optical action potential measurements were analyzed using custom KIC data analysis software (KIC DAT).

### Single cell equations

The single cell FitzHugh-Nagumo equations,

$$\begin{array}{cc} \tau_{1}\;\frac{dV}{dt} & =V-\frac{1}{3}V^{3}-W+d+I \end{array}$$ (5)

$$\begin{array}{cc} \tau_{2}\;\frac{dW}{dt} & =V+a-bW \end{array}$$ (6)

were obtained from Eqs (1 and 2) by setting ∂^2^*V*/∂*x*^2^ = 0. The nullclines,

$$W=V-\frac{1}{3}V^{3}+d+I$$ (7)

V=bW-a (8)

were obtained by setting *dV*/*dt* = 0 and *dW*/*dt* = 0, respectively.

### Partial differential equations

The partial differential Eqs (1 and 2) were transformed into ordinary differential equations by discretizing space using the method of lines [34]. The spatial derivatives were approximated by the second-order central differences,

$$\begin{array}{c} \frac{\partial^{2}V_{i,j}}{\partial x^{2}}\approx \frac{V_{i,j-1}-2V_{i,j}+V_{i,j+1}}{dx^{2}} \end{array}$$ (9)

and

$$\begin{array}{c} \frac{\partial^{2}V_{i,j}}{\partial y^{2}}\approx \frac{V_{i-1,j}-2V_{i,j}+V_{i+1,j}}{dy^{2}}. \end{array}$$ (10)

The ordinary differential equations were integrated forward in time using version 2019a of the Brain Dynamics Toolbox [35, 36] in conjunction with the Matlab ode45 solver. The solver error tolerances were AbsTol = 1e-6 and RelTol = 1e-6.

### Boundary conditions

All simulations used Neumann (no flux) boundary conditions unless stated otherwise. The Neumann conditions were implemented by treating cells on the outside edge of the boundary as if they had the same membrane potential as cells on the inside edge. The spatial gradient across the boundary was thus forced to ∂*V*/∂*x* = 0. The spatial derivatives at the vertical edges,

$$\begin{array}{c} \frac{\partial^{2}V_{i,1}}{\partial x^{2}}\approx \frac{-V_{i,1}+V_{i,2}}{dx^{2}} \end{array}$$

$$\begin{array}{c} \frac{\partial^{2}V_{i,L}}{\partial x^{2}}\approx \frac{V_{i,L-1}-V_{i,L}}{dx^{2}} \end{array}$$

were thus derived from Eq (9) by substituting *V*_*i*,0_ = *V*_*i*,1_ and *V*_*i*,*L*_ = *V*_*i*,*L*+1_. Likewise, the spatial derivatives at the horizontal edges,

$$\begin{array}{c} \frac{\partial^{2}V_{1,j}}{\partial y^{2}}\approx \frac{-V_{1,j}+V_{2,j}}{dy^{2}} \end{array}$$

$$\begin{array}{c} \frac{\partial^{2}V_{L,j}}{\partial y^{2}}\approx \frac{V_{L-1,j}-V_{L,j}}{dy^{2}} \end{array}$$

were derived from Eq (10) by substituting *V*_0,*j*_ = *V*_1,*j*_ and *V*_*L*,*j*_ = *V*_*L*+1,*j*_.

### Tissue boundaries

A similar approach was applied to cells on the boundary of the annulus (Figs 6, 7, 8 and 9) where cells within the annulus were isolated from the tissue by imposing zero flux with their neighbors. In matrix notation, that was achieved by computing the spatial derivatives as

$$\begin{array}{c} V_{xx}=\frac{V_{E}-2V+V_{W}}{{dx}^{2}} \end{array}$$

and

$$\begin{array}{c} V_{yy}=\frac{V_{N}-2V+V_{S}}{{dx}^{2}} \end{array}$$

where **V**_**N**_,**V**_**S**_,**V**_**E**_,**V**_**W**_ are circular shifted copies (north, south, east, west) of **V**. Prior to computing **V**_**xx**_ and **V**_**yy**_, designated cells (*i*, *j*) in **V**_**N**_, **V**_**S**_, **V**_**E**_ and **V**_**W**_ were first overwritten with their corresponding values in **V** to impose the no-flux condition. For example,

$$\begin{array}{c} V_{N}\left(i,j\right)=V\left(i,j\right) \end{array}$$

where *i*, *j* are the indexes of the cells that were designated to receive no flux from their northern neighbors. Individual cells could thus be configured to have zero flux with any or all of their designated neighbors.

### Numerical continuation

Numerical continuation of the single cell model (Fig 3) was performed using the November 2017 release of the Core Continuation (CoCo) Toolbox [37]. The overall tolerance of the correction algorithm was TOL = 1e-6. The minimum step size of the continuation algorithm was h_min = 0.01. The number of discretization intervals for the collocation algorithm was NTST = 20. Those same tolerances were also used to follow the branch of Hopf points in the stability analysis of the reduced model (Fig 5) with the maximum step size being constrained to h_max = 0.1.

### Biophysical cell model

The membrane potential (*V*) of the Qu-Chung model [16] is defined by six ionic currents and an external stimulation current,

$$\frac{dV}{dt}=I_{n}-\left(I_{Na}+I_{CaL}+I_{K}+I_{K1}+I_{Kp}+I_{b}\right)$$

where *I*_*n*_ is the stimulus current, *I*_*Na*_ is the fast sodium current, *I*_*CaL*_ is the slow inward calcium current, *I*_*K*_ is the time-dependent potassium current, *I*_*K*1_ is the time-independent potassium current, *I*_*Kp*_ is the plateau potassium current and *I*_*b*_ is the background current. The ionic currents have Hodgkin-Huxley style gating variables (*h*, *m*, *j*, *d*, *f*, *x*). Specifically,

$$\begin{array}{cc} I_{Na} & =I_{Na,max}\;m^{3}\;h\;j\;\left(V-E_{Na}\right),\\ I_{CaL} & =I_{CaL,max}\;d\;f\;\left(V-E_{CaL}\right),\\ I_{K} & =I_{K,max}\;X_{i}\;x\;\left(V-E_{K}\right),\\ I_{K1} & =I_{K1,max}\;\sqrt{{\left[K\right]}_{o}/5.4}\;K1_{\infty }\;\left(V-E_{K1}\right),\\ I_{Kp} & =I_{Kp,max}\;K_{p}\;\left(V-E_{Kp}\right),\\ I_{b} & =I_{b,max}\;\left(V+59.87\right), \end{array}$$

with reversal potentials,

$$\begin{array}{cc} E_{Na} & =\frac{RT}{F}\ln(\frac{{\left[Na\right]}_{o}}{{\left[Na\right]}_{i}}),\\ E_{CaL} & =7.7-13.0287\;\ln(\frac{{\left[Ca\right]}_{i}}{{\left[Ca\right]}_{o}}),\\ E_{K} & =\frac{RT}{F}\ln(\frac{{\left[K\right]}_{o}+PNaK}{{\left[K\right]}_{i}+PNaK{\left[Na\right]}_{o}}),\\ E_{K1} & =\frac{RT}{F}\ln(\frac{{\left[K\right]}_{o}}{{\left[K\right]}_{i}}),\\ E_{Kp} & =E_{K1}. \end{array}$$

The uptake of intracellular calcium is defined as,

$$\begin{array}{c} \frac{d{\left[Ca\right]}_{i}}{dt}=-0.0001\;I_{CaL}+0.07\;(0.0001-{\left[Ca\right]}_{i}). \end{array}$$

Each gating variable has kinetics of the form,

$$\begin{array}{c} \tau_{y}\frac{dy}{dt}=y_{\infty }-y, \end{array}$$

where *y* represents the gating variable, *τ*_*y*_ = 1/(*α*_*y*_ + *β*_*y*_) is the time constant of gate activation and *y*_∞_ = *α*_*y*_/(*α*_*y*_+ *β*_*y*_) is the steady state of the gate. The kinetics are unchanged from the LR1 model [18] except that the time constant for *x* is scaled by *γ* and the steady states of *d* and *f* are defined as,

$$\begin{array}{c} d_{\infty }=\frac{1}{1+\exp(-\left(V+V_{0}\right)/\alpha )} \end{array}$$

and

$$\begin{array}{c} f_{\infty }=\frac{1}{1+\exp(-\left(V+V_{0}+\Delta \right)/\beta )}, \end{array}$$

as described in [16]. The parameter values (Table 1) are unchanged from [16] except that we used *I*_*K*1,*max*_ = 0.3 instead of 0.6047.

**Table 1:** Parameters of the Qu-Chung model [16].

Parameter	Description
[*K*]_*o*_ = 5.4	Extracellular potassium (mM)
[*K*]_*i*_ = 145	Intracellular potassium (mM)
[*Na*]_*o*_ = 140	Extracellular sodium (mM)
[*Na*]_*i*_ = 10	Intracellular sodium (mM)
[*Ca*]_*o*_ = 1.8	Extracellular calcium (mM)
*R* = 8.314	Gas constant (J/K)
*T* = 310	Temperature (K)
*F* = 96.5	Faraday’s constant (C/mM)
*PNaK* = 0.0183	Maximal permeability of the NaK pump
*I*_*CaL*,*max*_ = 0.09	Maximal conductance of *I*_*CaL*_
*I*_*KP*,*max*_ = 0.0183	Maximal conductance of *I*_*Kp*_
*I*_*b*,*max*_ = 0.0392	Maximal conductance of *I*_*b*_
*I*_*Na*,*max*_ = 16	Maximal conductance of *I*_*Na*_
*I*_*K*1,*max*_ = 0.3	Maximal conductance of *I*_*K*1_
*I*_*K*,*max*_ = 0.282	Maximal conductance of *I*_*K*_
*γ* = 1 or 4	*τ*_*x*_ scaling factor
*α* = 9.4	*d*_∞_ slope parameter
*β* = 7.2	*f*_∞_ slope parameter
Δ = 0 or -15	*f*_∞_ shift parameter (mV)
*V*_0_ = 24.5	Center of the *I*_*CaL*_ activation window (mV).

## Supporting information

S1 Video Effect of mixing monostable and bistable cells in the same tissue. Animated version of Fig 4.
(MP4)

S2 Video Rogue activity emerges preferentially at tissue boundaries. Animated version of Fig 6.
(MP4)

S3 Video Ectopic activity induced at the tissue boundary by a passing wave. Animated version of Fig 8.
(MP4)

S4 Video Pathways for the progression of disease. Animated version of Fig 9.
(MP4)

S5 Video Effect of mixing monostable and bistable cells in a biophysical model. Animated version of Fig 11.
(MP4)
